# Aspirin Inhibits Natural Killer/T-Cell Lymphoma by Modulation of VEGF Expression and Mitochondrial Function

**DOI:** 10.3389/fonc.2018.00679

**Published:** 2019-01-14

**Authors:** Hongyu Zhang, Jianping Lu, Yun Jiao, Qi Chen, Min Li, Zichen Wang, Zhendong Yu, Xiaodong Huang, Athena Yao, Qiong Gao, Weiguo Xie, Ling Li, Paul Yao

**Affiliations:** ^1^Department of Hematology, Peking University Shenzhen Hospital, Shenzhen, China; ^2^Department of Child Psychiatry, Kangning Hospital of Shenzhen, Shenzhen, China; ^3^Department of Pediatrics, Hainan Maternal and Child Health Hospital, Haikou, China; ^4^Institute of Rehabilitation Center, Tongren Hospital of Wuhan University, Wuhan, China; ^5^Department of Gynecology, The Eighth Affiliated Hospital, Sun Yat-sen University, Shenzhen, China

**Keywords:** aspirin, epstein-barr virus, mitochondria, NKTCL, reactive oxygen species

## Abstract

Extranodal nasal-type natural killer/T-cell lymphoma (NKTCL) is an Epstein-Barr virus (EBV)-associated lymphoma with a strong tendency relapse or be refractory in response to chemotherapy. Development of a new strategy for NKTCL treatment is still quite necessary. In this study, we found that aspirin treatment suppresses VEGF expression in NKTCL SNK-6 cells. Further investigation showed that aspirin treatment increases histone methylation in the range of −100~0 that is proximal to the transcription start site on the VEGF promoter, subsequently decreasing the binding ability of Sp1 to the VEGF promoter with VEGF suppression. Furthermore, aspirin treatment modulates mitochondrial function with increased ROS formation and apoptosis in NKTCL cells. Aspirin treatment alone slightly inhibits NKTCL SNK-6 tumor growth and EBV replication; while in the presence of histone deacetylase inhibitor (HDACi) chidamide (CDM), aspirin significantly suppresses the VEGF signaling pathway with increased ROS overgeneration and EBV inhibition. We also showed that with the addition of chidamide, aspirin significantly suppresses NKTCL tumor growth in both *in vitro* cell culture and *in vivo* mouse model with prolonged mouse survival. This is the first time that the potential mechanism for aspirin-mediated VEGF suppression and anti-tumor effect has been discovered, and this study provides a new strategy for anti-tumor drug development for NKTCL treatment based on aspirin-mediated targeting of the VEGF signaling pathway and ROS formation.

## Introduction

Extranodal nasal-type natural killer/T-cell lymphoma (NKTCL) is a rare subtype of lymphoma that develops primarily in the nasal cavity or in extranasal sites. It is characterized as an aggressive disease with frequent deletions on chromosome 6q.3-14 (1). NKTCL is closely associated with Epstein-Barr virus (EBV) infection (2). It has NK/T-cell markers (CD3 and CD56) and cytotoxic molecules and is characterized by angiocentric and invasive lymphoma cell infiltration and aggressive necrotic lesions in the nasal cavity and palate. Epidemiological study shows that NKTCL is dominant in Asia and Latin America but is very rare in Western countries. NKTCL occurs mostly in younger patients and often is characterized by frequent local progression, but also tends to have extranodal dissemination. Radiotherapy is considered to be the main treatment for early-stage NKTCL and has relatively good outcomes, but the frequent relapse/refractory tendencies of NKTCL may result in a poor prognosis. Therefore, development of a new strategy for the treatment of NKTCL is still quite necessary (1, 3, 4).

Aspirin (acetylsalicylic acid, ASA), a type of widely used non-steroidal anti-inflammatory drug, has been found to be an effective agent against many human cancers, including colorectal cancer (CRC) (5–7), leukemia (8), and breast cancer (9). Regular use of aspirin can result in improved survival of many cancers, although the effect is relatively weak and has significant side effects (5, 7, 10). Many potential targets and mechanisms of aspirin have been reported, including heparanase (11), epigenetics (12), NFκB (13), CDH1 (14), and the mitochondria (15), although the detailed mechanism is still largely unknown (16).

Chidamide (CDM, CS055) is a novel benzamide-type histone deacetylase inhibitor (HDACi) (17), a synthetic analog of MS-275 (18), and is currently used for treatment of leukemia (19). We have recently found that CDM inhibits EBV replication through overgeneration of ROS (reactive oxygen species) in EBV-associated tumors (20), and that it also increases p300 over-acetylation in acute myeloid leukemia (AML) cells with dissociation of p300 from HIF1α, subsequently suppressing the HIF1α/VEGF pathway (21). We suppose that CDM may suppress EBV-associated NKTCL tumor growth by inhibition of the HIF1α/VEGF pathway and ROS overgeneration.

In an effort to improve the pharmacological properties of aspirin, we investigated the potential effect and mechanism of aspirin in NKTCL cells. We found that aspirin suppresses VEGF expression through histone methylation and the subsequent decreased association of Sp1 on the VEGF promoter. Furthermore, it modulates mitochondrial function and ROS generation, leading to increased apoptosis in SNK-6 cells. Aspirin treatment alone slightly suppressed tumor growth, while with the addition of histone deacetylase inhibitor chidamide, aspirin significantly potentiated the effect on NKTCL tumor suppression by decreasing VEGF expression and EBV replication, prolonging mouse survival. This is the first time a new aspirin-based strategy has been developed for the treatment of NKTCL with the addition of HDACi chidamide.

## Materials and Methods

### Reagents and Materials

The NKTCL cell lines, including HANK-1, NK-92, SNT-8, and SNK-6 cells, were purchased from ATCC and cultured in RPMI 1640 medium containing 2 mmol/l glutamine supplemented with 100 U/ml penicillin, 100 μg/ml streptomycin, 10% human serum and 1,000 U/ml recombinant human IL-2. All cells were maintained in a humidified incubator with 5% CO_2_ at 37°C.

Antibodies for β-actin (sc-47778), C/EBPα (sc-7962), EBV Ea-D (sc-58121), EBV ZEBRA (BZ1, sc-53904), Sp1 (sc-17824) and VEGF (sc-7269) were obtained from Santa Cruz Biotechnology. Antibodies for acetyl-histone H4 K5, K8, K12, and K16 (H4K5,8,12,16ac, #PA5-40084) were obtained from Invitrogen. Antibodies for anti-histone H3 acetyl K9, K14, K18, K23, K27(H3K9,14,18,23,27ac, ab47915), H4K20me1 (ab9051), H4K20me3 (ab9053), H4R3me1 (ab17339), H3K9me2 (ab1220), H3K9me3 (ab8898), and H3K27me3 (ab6002), H2AX (ab20669) and γH2AX (ab2893) were obtained from Abcam, 3-nitrotyrosine (3-NT) was measured by 3-Nitrotyrosine ELISA Kit (ab116691 from Abcam). Nuclear extracts were prepared using the NE-PER Nuclear and Cytoplasmic Extraction Reagents Kit (Pierce Biotechnology). Protein concentration was measured using the Coomassie Protein Assay Kit (Pierce Biotechnology) per manufacturers' instructions. The siRNA for Sp1 (# 4457308), ENX-1 (# 4392420) and negative control (# AM4636) were obtained from Ambion and transfected by Lipofectamine® 2000 Reagent (Invitrogen). Luciferase activity assay was carried out using the Dual-Luciferase™ Assay System (Promega) and the transfection efficiency was normalized using a cotransfected renilla plasmid.

Acetylsalicylic acid (Aspirin, ASA, #A5376) was obtained from Sigma. PRC2 specific inhibitor EDD266 (#2083627-02-3) was obtained from ChemScene llc. Chidamide (CDM, CS055) was supplied by Chipscreen Biosciences (Shenzhen, China) and was dissolved in DMF (dimethyl-formamide). For the *in vivo* experiments, CDM was suspended in 0.1% sodium carboxyl methylcellulose and stored at 4°C.

### Construction of Plasmids and Vectors

The human genomic DNA was prepared from the SNK-6 cells. In order to construct the VEGF reporter plasmid, the VEGF gene promoter (Ensembl gene ID: VEGFA-201 ENST00000230480.10) was amplified by PCR and subcloned into the pGL3-basic vector (# E1751, Promega) using restriction sites of Mlu I and Hind III with the following primers: Forward: 5′-gcgc-acgcgt- ctg tga acc ttg gtg ggg gtc−3′ (Mlu I) and Reverse: 5′- gtac- aagctt- ctc gag agg tca cct tcc cgc−3′ (Hind III). To map VEGF promoter activity, the related deletion promoter constructs were generated by PCR methods and subcloned into the pGL3-basic vector. In order to construct the Sp1 expression plasmid, the human Sp1 cDNA (from Open Biosystems) was amplified by PCR and subcloned into pcDNA3.1 using restriction sites of HindIII and XhoI with the following primers: Forward: 5′- gtac- aagctt- atg agc gac caa gat cac tcc−3′ (Hind III) and Reverse: 5′-gcgc- ctcgag - tca gaa gcc att gcc act gat−3′ (XhoI). All the vectors were verified by sequencing, and detailed information on these plasmids is available upon request (21).

### RT Reaction and Real-Time Quantitative PCR

Total RNA from treated cells was extracted using the RNeasy Micro Kit (Qiagen), and the RNA was reverse transcribed using an Omniscript RT kit (Qiagen). All the primers were designed using Primer 3 Plus software with the Tm at 60°C, primer size of 21 bp, and the product length in the range of 140-160 bp (see Table S1). The primers were validated with the amplification efficiency in the range of 1.9–2.1, and the amplified products were confirmed with agarose gel. Real-time quantitative PCR was run on iCycler iQ (Bio-Rad) with the Quantitect SYBR green PCR kit (Qiagen). The PCR was performed by denaturing at 95°C for 8 min, followed by 45 cycles of denaturation at 95°C, annealing at 60°C, and extension at 72°C for 10 s, respectively. 1 μl of each cDNA was used to measure target genes. β-actin was used as the housekeeping gene for transcript normalization, and the mean values were used to calculate relative transcript levels with the ^ΔΔ^CT method per instructions from Qiagen. In brief, the amplified transcripts were quantified by the comparative threshold cycle method using β-actin as a normalizer. Fold changes in gene mRNA expression were calculated as 2^−ΔΔ*CT*^ with CT = threshold cycle, ΔCT = CT (target gene)-CT(β-actin), and the ΔΔCT = ΔCT (experimental)-ΔCT (reference) (21, 22).

### Western Blotting

Cells were lysed in an ice-cold lysis buffer (0.137 M NaCl, 2 mM EDTA, 10% glycerol, 1% NP-40, 20 mM Tris base, pH 8.0) with protease inhibitor cocktail (Sigma). The proteins were separated in 10% SDS-PAGE and further transferred to the PVDF membrane. The membrane was incubated with appropriate antibodies, washed and incubated with HRP-labeled secondary antibodies, and then the blots were visualized using the ECL+plus Western Blotting Detection System (Amersham). The blots were quantitated by IMAGEQUANT, and final results were normalized by β-actin (21, 22).

### Luciferase Reporter Assay

1.0 × 10^5^ of SNK-6 cells were seeded in a 6-well plate with complete medium to grow until they reached 80% confluence. Cells were then cotransfected by 3 μg of VEGF full length or deletion reporter constructs, together with 0.2 μg of pRL-CMV-Luc *Renilla* plasmid (from Promega). Then, cells were treated by either 5 mM aspirin or empty control (CTL) for 24 h. After treatment, the cells were harvested and the luciferase activity assays were carried out using the Dual-Luciferase^TM^ Assay System (Promega), and the transfection efficiencies were normalized using a cotransfected *Renilla* plasmid according to manufacturers' instructions. The VEGF reporter activity from either ASA or control (CTL) was calculated (21).

### Chromatin Immunoprecipitation (ChIP)

Cells were washed and crosslinked using 1% formaldehyde for 20 min and terminated by 0.1M glycine. Cell lysates were sonicated and centrifuged. Five Hundred micrograms of protein were pre-cleared by BSA/salmon sperm DNA with preimmune IgG and a slurry of Protein A Agarose beads. Immunoprecipitations were performed with the indicated antibodies, BSA/salmon sperm DNA and a 50% slurry of Protein A agarose beads. Input and immunoprecipitates were washed and eluted, then incubated with 0.2 mg/ml Proteinase K for 2 h at 42°C, followed by 6 h at 65°C to reverse the formaldehyde crosslinking. DNA fragments were recovered through phenol/chloroform extraction and ethanol precipitation. A ~150 bp fragment in the range of −200~0 from the transcription start site on the VEGF promoter was amplified by real-time PCR (qPCR) using the primers provided in Table S1 (21, 22).

### Immunostaining

The treated SNK-6 cells were transferred to cover slips coated with 0.1% gelatin, fixed by 3.7% formaldehyde at 37°C for 15 min, permeabilized by 1% BSA+0.2% Triton X-100 in PBS for 1 h, and then blotted with 40 μg/ml (dilute 1:50) of Ki-67 (MIB-1) mouse monoclonal antibody for 2 h. The cells were then washed three times and the FITC labeled anti-mouse secondary antibody (1:100) was added for blotting for another 1 h. After thorough washing, the slides were visualized and photographed, and the nuclei of cells were stained with 4',6-diamidino-2-phenylindole dihydrochloride (DAPI, #D9542, from Sigma), and the positive Ki-67 cells were quantitated.

### Measurement of ROS Generation

Treated cells were seeded in a 24-well plate and incubated with 10 μM CM-H2DCFDA (Invitrogen) for 45 min at 37°C, and then the intracellular formation of reactive oxygen species (ROS) was measured at excitation/emission wavelengths of 485/530 nm using a FLx800 microplate fluorescence reader (Bio-Tek). The data was normalized as arbitrary units (21, 23).

### Measurement of DNA Breaks

8-OHdG formation was measured using an OxiSelect™ Oxidative DNA Damage ELISA Kit (Cat No. STA320, from Cell Biolabs Inc.) per manufacturers' instructions. The formation of γH2AX was measured from nuclear extracts by western blotting using H2AX as the input control (21).

### Measurement of Apoptosis

Apoptosis was evaluated by TUNEL assay using the *In Situ* Cell Death Detection Kit™ (Roche). Cells were fixed in 4% paraformaldehyde and labeled with TUNEL reagents. Stained cells were photographed by a fluorescence microscope and further quantified by FACS analysis. Caspase-3 activity was determined using the ApoAlert caspase assay kit (Clontech). Treated cells were harvested and 50 μg of proteins were incubated with the fluorogenic peptide substrate Ac-DEVD-7-amino-4-trifluoromethyl coumarin (AFC). The initial rate of free AFC release was measured using a FL × 800 microplate reader (Bio-Tek) at excitation/emission wavelengths of 380/505 nm, and enzyme activity was calculated as pmol/min/mg (23).

### Measurement of Mitochondrial Function

Intracellular ATP level was determined using the luciferin/luciferase-induced bioluminescence system. An ATP standard curve was generated at concentrations of 10^−12^-10^−3^M, and intracellular ATP levels were calculated and expressed as nmol/mg protein. Mitochondrial membrane potential (Δψm) was measured using TMRE (from Molecular Probes T-669) staining. A 600 μM T-669 stock solution was prepared using DMSO. Cells were grown on coverslips and immersed in 600 nM TMRE for 20 min at 37°C to load them with dye. The labeling medium was then aspirated and the cells were immersed in 150 nM TMRE to maintain an equal distribution of the fluorophore. The coverslips were mounted with live cells onto confocal microscopes to image the cells using 548 nm excitation/573 nm emission filters, and the intensity of TMRE fluorescence was measured using Image J software. Data from 10 to 20 cells were collected for each experimental condition and mean values of fluorescence intensity ± SEM were calculated (24).

### Detection of EBV Copy Number

Genomic DNA was extracted from treated SNK-6 cells or SNK-6 tumor tissue in mice using a QIAamp DNA Mini Kit (Qiagen). The EBV DNA copy number was measured through qPCR using 50 ng of total DNA with EBV BMRF1 primers (see Table S1), and the results were normalized using cellular β-actin (primers see Table S1) as an internal control (25, 26). The Namalwa cell line, which contains 2 EBV viral genome copies, was used as a standard to prepare calibration curves for both EBV BMRF1 and β-actin genes, and the EBV viral load was presented as the number of viral genomes per cell (27, 28).

### Cell Viability and MTT Assay

Cells were pooled in 12-well plates following exposure to different treatments as indicated at 80% confluence. Cell viability was analyzed using the MTT (3-(4,5-dimethylthianol-2-yl)- 2,5 diphenyltetrazolium bromide) reduction assay (29). In brief, the cells in each well were aspirated and washed with PBS, and then 0.2 ml of 0.3 mg/ml MTT solution were added at 25°C for 3 h. Thereafter, the precipitated blue formazan product was extracted by incubating samples with 0.1 ml 10% SDS (dissolved by 0.01M HCl) overnight at 37°C. The optical density (OD) of formazan concentrations was determined at 560 nm and the background was subtracted at 670 nm, then normalized by cell numbers, and expressed as OD/10^6^ cells (21, 23).

### DNA Synthesis by [^3^H]-Thymidine Incorporation

Cell proliferation was evaluated as the rate of DNA synthesis by [^3^H]-methylthymidine incorporation (30). Cells were pooled in 24-well plates until they reached 80% confluence, and then the indicated chemicals were added and incubated for 24 h. At the end of the treatment, cells were incubated with serum-free media containing ^3^H-methylthymidine (0.5 μCi/well) for 2 h and then washed twice with PBS. Cellular DNA was precipitated using 10% trichloroacetic acid and solubilized with 0.4 M NaOH (0.5 ml/well). Incorporation of ^3^H-methylthymidine into the DNA was measured in a scintillation counter and was determined as counts per minute (CPM) (21).

### Colony Formation in Soft Agar

This assay is a method for evaluating the ability of individual cell lines to grow in an anchorage-independent manner. Cells were resuspended in DMEM containing 5% FBS with 0.3% agarose and layered on top of 0.5% agarose in DMEM on 60-mm plates. One thousands of cells were seeded in 60 mm soft agar dishes for 30 days. The dishes were examined twice per week, and colonies that grew beyond 50 mm in diameter were scored as positive. Each experiment was done in quadruplicate (21).

### Migration and Invasion Assays

Cell migration and invasion assays were performed in 24-well chemotaxis plates with an 8 μm polycarbonate filter membrane. The plates were coated with 20 μg Matrigel for invasion assays and uncoated for migration assays. Invasion and migration were expressed as the number of migrated cells bound per microscopic field and averaged from at least four fields per assay in at least 4 experiments (31, 32).

### Animals

Balb/c athymic nude male mice (6 weeks old) were obtained from the Guangdong Medical Animal Center. All procedures involving mice were conducted in accordance with NIH regulations concerning the use and care of experimental animals, and were approved by the Institutional Animal Care and Use Committee (from Peking University Shenzhen Hospital). The 2 × 10^6^ viable treated tumor cells were washed, harvested in PBS, and then injected into the lateral tail vein in a volume of 0.1 ml. Two days after the implantation of the primary xenograft, the mice were treated with either aspirin (ASA) or 25 mg/kg of body mass of CDM (0.1% sodium carboxyl methylcellulose as vehicle), or a combination of ASA/CDM via oral gavage 3 times a week. The experimental mice were separated into 5 groups (n = 9) as follows. Group 1 (CTL): Chemical vehicle (0.1% sodium carboxyl methylcellulose) as control; Group 2 (ASA-100): Low-dose of aspirin (100 mg/kg); Group 3 (ASA-400): High-dose of aspirin (400 mg/kg); Group 4: (ASA-100/CDM): Low-dose of aspirin (100 mg/kg) plus CDM (25 mg/kg in 0.1% sodium carboxyl methylcellulose); Group 5 (ASA-400): High-dose of aspirin (400 mg/kg) plus CDM (25 mg/kg). Mice were monitored for changes in body weight and sacrificed when values fell below 20% of the initial weight. The lungs of sacrificed mice were isolated and fixed in 10% formalin. The number of surface metastases per lung was determined under a dissecting microscope. Formalin-fixed, paraffin-embedded tumor tissue from the lungs were sectioned to 4 mm thickness, and the histopathological analyses were performed with H&E staining. Images were taken using a Carl Zeiss MIRAX MIDI slide scanner, and analyses were performed using a 3DHISTECH Pannoramic Viewer. The tumor tissues were isolated for *in vivo* monitoring of superoxide anion release and gene expression of tumor tissues were measured through real time PCR for mRNA and Western Blotting for protein levels (21).

### *In vivo* Superoxide Release

Superoxide anion (O${}_{2}^{.-}$) release from tumor tissues was determined using a luminol-EDTA-Fe enhanced chemiluminescence (CL) system supplemented with DMSO-TBAC (Dimethyl sulfoxide-tetrabutyl-ammonium chloride) solution for extraction of released O${}_{2}^{.-}$ from tissues, as described previously (23). Superoxide levels were calculated from the standard curve generated by the xanthine/xanthine oxidase reaction (21).

### Statistical Analysis

The data was given as mean ± SEM; all of the experiments were performed at least in quadruplicate unless otherwise indicated. One-way ANOVA followed by the Bonferroni *post hoc* test was used to determine statistical significance of different groups. The mouse survival curve was determined through Kaplan-Meier survival analysis using SPSS 22 software, and a *P* < 0.05 was considered significant (21).

## Results

### Aspirin Treatment Suppresses VEGF Expression in NKTCL Cells

**Figure 1:**
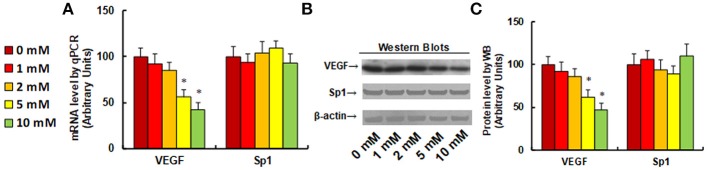
Aspirin treatment suppresses VEGF expression in SNK-6 cells. SNK-6 cells were treated by ASA with different concentrations of 0, 1, 2, 5, and 10 mM for 24 h, and then the cells were used for the analysis of gene expression. **(A)** mRNA level by qPCR, *n* = 4. **(B)** Representative pictures for Western Blotting. **(C)** Quantitation of protein levels for **(B)**, *n* = 5. ^*^P < 0.05, vs. CTL group. Data are expressed as mean ± SEM.

It has been previously reported that aspirin inhibits VEGF and tumor growth by direct downregulation of Sp1 in colon cancer cells (33). In this study, we evaluated the effect of aspirin on the gene expression of VEGF and Sp1 in different kinds of NKTCL cells. In Figure 1, the SNK-6 cells were treated with different concentrations of aspirin: 0, 1, 2, 5, and 10 mM. The results showed that 1 and 2 mM of aspirin had no effect, while 5 and 10 mM of aspirin decreased VEGF mRNA by 46 and 58%, respectively. On the other hand, there was no difference in Sp1 expression (see Figure 1A). We then measured the effect of aspirin on the protein levels of VEGF and Sp1. The results showed that 1 and 2 mM of aspirin had no effects on protein expression, while 5 and 10 mM of aspirin suppressed VEGF expression by 38 and 53%, respectively, and Sp1 had no effect. Then, we measured the effect of aspirin on HANK-1 (see Figure S1A), NK-92 (see Figure S1B) and SNT-8 cells (see Figure S1C), and found that aspirin had no effect on Sp1 expression, while 5 and 10 mM of aspirin treatment significantly suppressed VEGF expression. These results indicate that aspirin treatment suppresses VEGF expression in NKTCL cells, but does not suppress Sp1 expression.

### Aspirin Regulates VEGF Expression Through Histone Methylation and Subsequently Decreased Association of Sp1 on the VEGF Promoter

**Figure 2:**
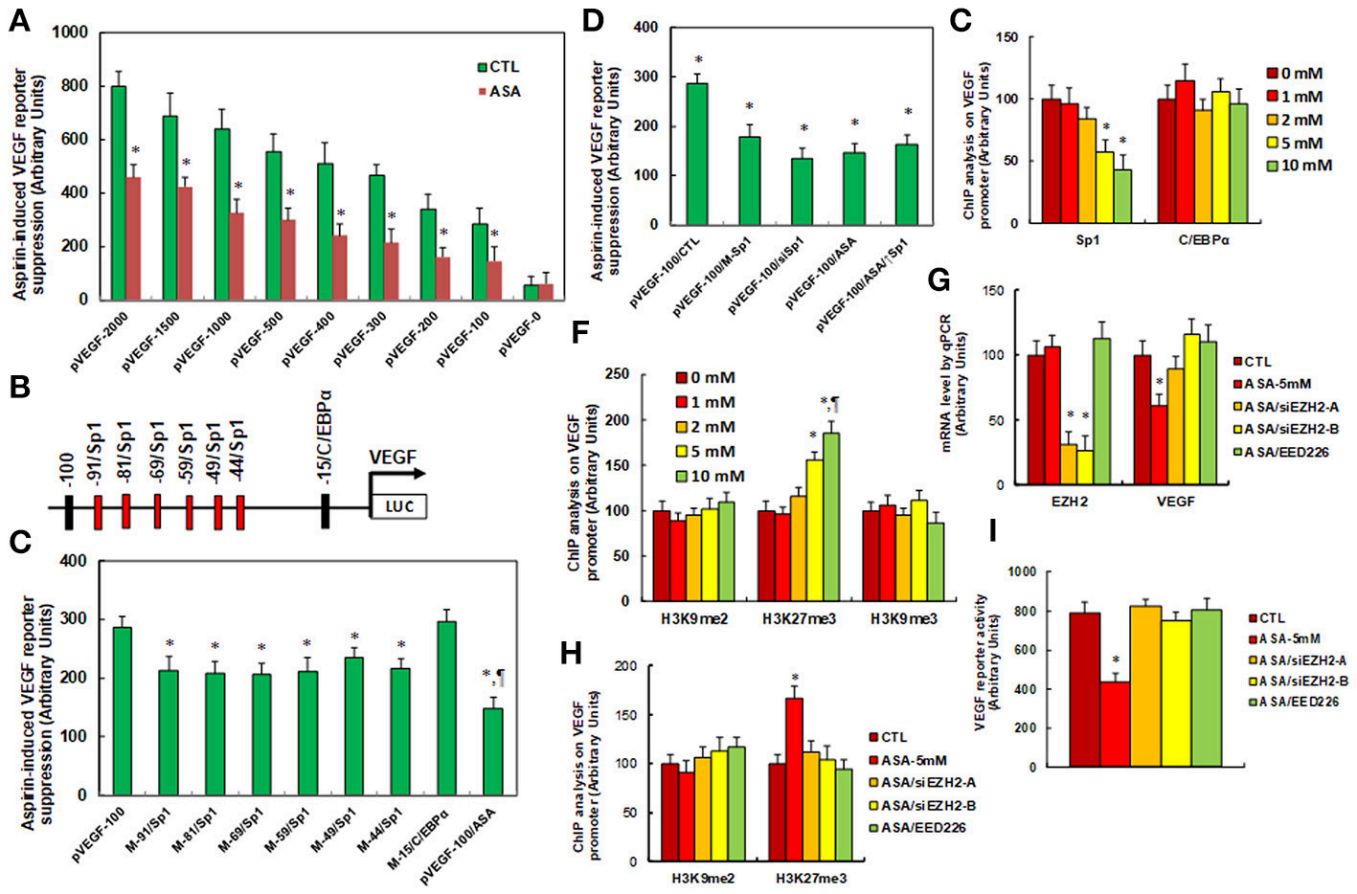
Aspirin regulates VEGF expression through histone methylation and the subsequent decreased association of Sp1 on the VEGF promoter**. (A)** The SNK-6 cells were transiently transfected by either VEGF full length (pVEGF-2000) or deletion reporter plasmids. After 24 h, the cells were treated by either control (CTL) or 5 mM ASA for 24 h, and the VEGF reporter activities were calculated, *n* = 4. ^*^P < 0.05, vs. pVEGF-2000 group, *n* = 4. **(B)** The schematic picture for the potential transcriptional binding motif in the range of −100~0 (from transcription start site) on the VEGF promoter in addition to the 6 potential Sp1 binding sites are marked in red. **(C)** The SNK-6 cells were transiently transfected by either wild type VEGF deletion (−100) reporter construct (pVEGF-100), or single point mutation of Sp1 and C/EBPα at the site shown in **(B)**, then treated with either control or 5 mM ASA for 24 h, and the VEGF reporter activities were calculated. **(D)** The SNK-6 cells were transiently transfected by either CTL (transfected with control siRNA plus empty luciferase vector), or wild type VEGF deletion (−100) reporter construct (pVEGF-100), or single point mutation of 6 Sp1 sites in one reporter (pVEGF-100/M-Sp1), and then treated with either siSp1(pVEGF-100/siSp1), or 5 mM ASA (pVEGF-100/ASA) alone, or ASA together Sp1 overexpression plasmid (pVEGF-100/ASA/↑Sp1) for 24 h and the VEGF reporter activities were calculated. **(E)** SNK-6 cells SNK-6 cells were treated by ASA with different concentrations of 0, 1, 2, 5, and 10 mM for 24 h, then the cells were used for ChIP analysis by Sp1 and C/EBPα antibodies, and the VEGF promoter in the range of −200~0 was amplified and measured by qPCR, *n* = 5. ^*^P < 0.05, vs. 0 mM group. **(F)** SNK-6 cells were used for ChIP analysis by H3K9me2, H3K27me3, and H3K9me3 antibody, respectively, and the VEGF promoter in the range of −200~0 was amplified and measured by qPCR, *n* = 5. **(G–I)** The SNK-6 cells were treated by control (CTL), 5 mM aspirin (ASA-5 mM), aspirin with EZH2 siRNA cell line **(A,B)** (ASA/siEZH2-B), or 2μM of EED266 for 24 h, and the cells were harvested for further analysis. **(G)** mRNA levels by qPCR for EZH2, EED, and VEGF, *n* = 4. **(H)** ChIP analysis by H3K9me2 and H3K27me3, respectively on the VEGF promoter, *n* = 5. **(I)** VEGF reporter activity assay, *n* = 5. ^*^P < 0.05, vs. 0mM group; ^¶^*P* < 0.05, vs. 5mM group. Results are expressed as mean ± SEM.

We investigated the potential molecular mechanism for aspirin-mediated VEGF suppression. A series of progressive 5'-promoter deletion constructs for the VEGF promoter were generated, and these constructs were transfected into SNK-6 cells for the analysis of VEGF reporter activity in the presence of 5 mM aspirin (ASA). We found that VEGF-induced reporter suppression occurred among the −2000, −1500, −1000, −500, −400, −300, −200 and −100 deletion constructs (numbered according to Ensembl gene ID: VEGFA-201 ENST00000230480.10, transcription start site was marked as 0), while activity did not decrease in the pVEGF-0 deletion reporter construct, indicating that aspirin-responsive transcriptional element is located in the range of −100~0 on the VEGF promoter (see Figure 2A). The transcription factor databases TESS revealed many potential binding motifs, including 1 of C/EBPα and 6 of Sp1 binding sites (marked in red) located at the range of −100~0 on the VEGF promoter (see Figure 2B). We then mutated these potential binding motifs in the−100 deletion reporter construct (see Figure 2C), and VEGF mutation reporter assay showed that mutation of any Sp1 binding motif at either −91, −81, −69, −59, −49, or −44 significantly decreased pVEGF-100 reporter activity compared to the pVEGF-100 wild type reporter. This partly mimicked the effect of aspirin-induced VEGF suppression, while mutation of C/EBPα at−15 did not show any effects (see Figure 2C). We then mutated all of the 6 Sp1 binding sites in one pVEGF-100 construct (pVEGF-100/M-Sp1), and the cells were transfected with pVEGF-100 wild type (pVEGF-100), pVEGF-100/M-Sp1, pVEGF-100 wild type with siRNA for Sp1(pVEGF-100/siSp1), pVEGF-100 with 5 mM ASA (pVEGF-100/ASA), or pVEGF plus 5 mM ASA together with the Sp1 overexpression plasmid (pVEGF-100/ASA/↑Sp1) for 24 h. Our results showed that mutation of 6 of the Sp1 binding sites (pVEGF-100/M-Sp1) significantly decreased pVEGF-100 reporter activity and resulted in reporter activity similar to that of the treatment of pVEGF-100/ASA and pVEGF-100/siSp1. In addition, ASA-induced pVEGF-100 reporter suppression (pVEGF-100/ASA) could not be restored by Sp1 overexpression (pVEGF-100/ASA/↑Sp1), indicating that aspirin-induced VEGF suppression may be due to ASA-induced dissociation of Sp1 in the range of −100~0 on the VEGF promoter (see Figure 2D). We then conducted ChIP analysis using antibodies of Sp1 and C/EBPα (see Figure 2E); the results showed that Sp1 binding ability on the VEGF promoter was significantly decreased in the treatments of both the 5 and the 10 mM ASA groups, while C/EBPα binding ability did not change (see Figure 2E). Given the fact that Sp1 protein expression levels did not change during ASA treatment, ASA-induced decreased association of Sp1 on the VEGF promoter may be due to ASA-induced epigenetic changes in the range of −100~0 on the VEGF promoter. We first evaluated histone acetylation on the VEGF promoter using the acetyl-histone H4 (K5, K8, K12, K16) antibody that recognizes histone H4 acetylated at lysines 5, 8, 12, or 16 and the acetyl-histone H3 (K9, K14, K18, K23, K27) antibody that recognizes histone H3 acetylated at lysines 9, 14, 18, 23, or 27 by ChIP analysis (see Figure S2A). The results showed that there was no significant difference in either histone H3 or H4 acetylation. We then measured histone methylation on the VEGF promoter. We first evaluated histone H4 methylation on the VEGF promoter (see Figure S2B) and found that aspirin did not have any effect on histone H4 methylation. We then evaluated the effect of aspirin on histone H3 methylation (see Figure 2F). The results showed that aspirin treatment had no effect on the methylation of H3K9me2 or H3K9me3, while methylation of H3K27me3 increased to 156 and 186% by 5 and 10 mM aspirin treatment respectively. In order to further confirm that VEGF suppression is due to aspirin-induced H3K27me3 modification on the VEGF promoter, the SNK-6 cells were treated by either siRNA for EZH2, a catalytic subunit of PRC2 that is responsible for H3K27me3 methylation (34), or PRC2 specific inhibitor EED226 (34), and the cells were harvested for further analysis. We first measured mRNA expression. The results showed that EZH2 mRNA expression was decreased to 31 and 26%, respectively, in EZH2 siRNA cell line A (ASA/siEZH2-A) and B (ASA/siEZH2-B) compared to the control (CTL) group, indicating a successful knockdown by EZH2 siRNA for SNK-6 cell line A and B. EED mRNA expression did not change in response to the different treatments. Additionally, VEGF mRNA was decreased to 61% as a result of aspirin (ASA) treatment compared to the CTL group, while both EZH2 siRNA and PRC2 inhibitor EED226 treatment completely restored the effect of aspirin (see Figure 2G). We then measured histone methylation on the VEGF promoter using ChIP analysis (see Figure 2H). The results showed that H3K9me2 modification did not change in response to any of the treatments, while H3K27me3 modification increased to 167% in response to aspirin treatment (ASA) compared to the CTL group, and either the EZH2 siRNA treatments (ASA-siEZH2A or ASAsiEZH2-B), or EED226 treatment completely restored this effect. We finally measured VEGF reporter activity (see Figure 2I), and the results showed that treatments of either EZH2 siRNA or EED226 completely restored aspirin-induced decreased VEGF reporter activity. Our results indicate that aspirin-induced VEGF suppression in SNK-6 cells is due to aspirin-mediated H3K27me3 histone methylation and the subsequently dissociation of Sp1 on the VEGF promoter.

### Aspirin Treatment Modulates ROS Generation, DNA Damage, Mitochondrial Function and Apoptosis in SNK-6 Cells

**Figure 3:**
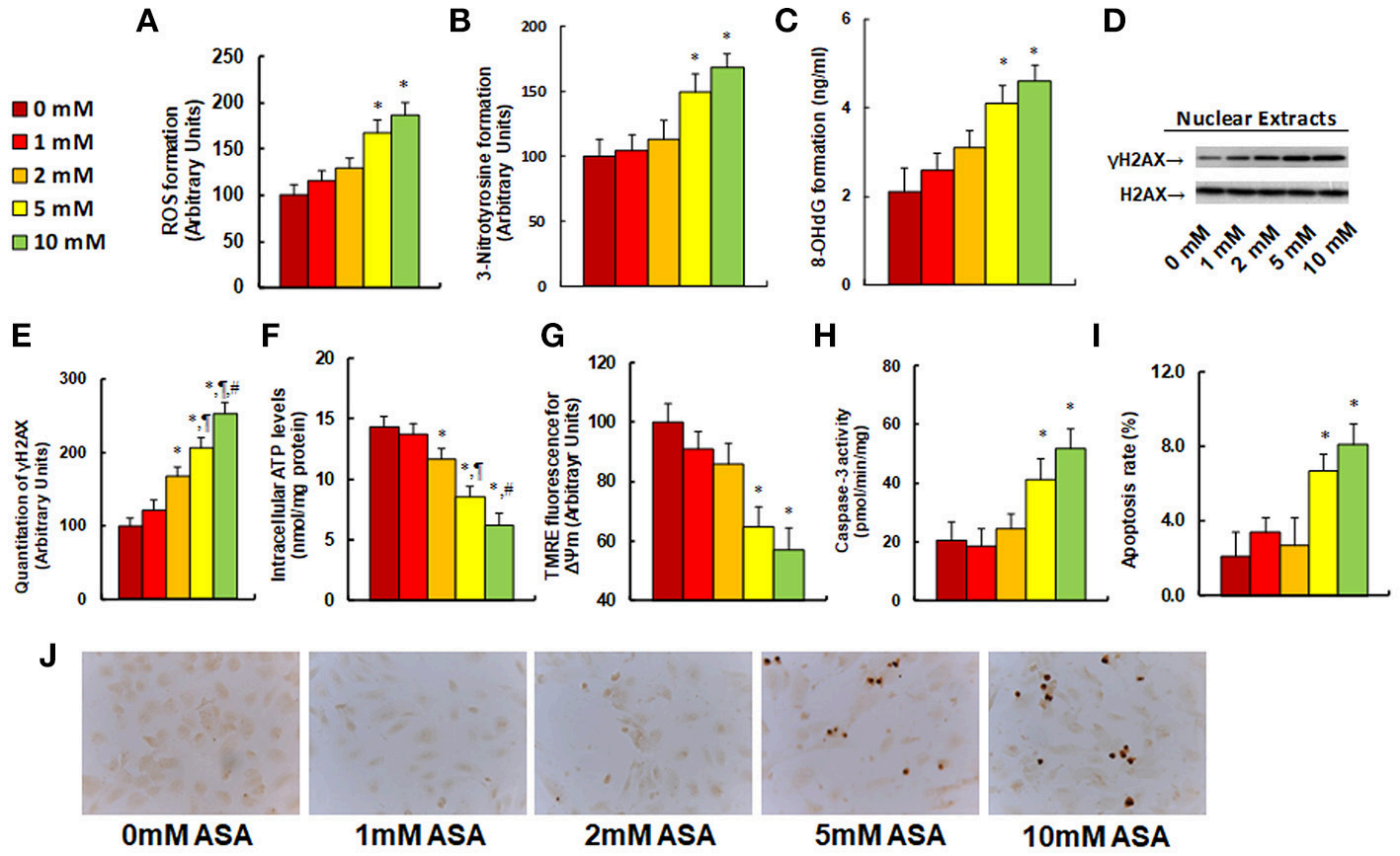
Aspirin treatment modulates ROS generation, DNA damage, mitochondrial function and apoptosis in SNK-6 cells. SNK-6 cells were treated by ASA with different concentrations of 0, 1, 2, 5, and 10 mM for 24 h, and then the cells were used for biomedical analysis. **(A)** ROS formation, *n* = 5. **(B)** 3-nitrotyrosine (3-NT) formation, *n* = 5. **(C)** 8-OHdG formation, *n* = 5. **(D)** Representative western blotting bands for γH2AX. **(E)** Quantitation of γH2AX formation for **(D)**, *n* = 5. **(F)** Intracellular ATP level, *n* = 5. **(G)** Mitochondrial membrane potential (ΔΨ m) by TMRE fluorescence, *n* = 5. **(H)** Caspase-3 activity, *n* = 5. **(I)** Apoptosis rate by TUNEL assay, *n* = 5. **(J)** Representative pictures for **(I)**. ^*^P < 0.05, vs. 0 mM group; ^¶^*P* < 0.05, vs. 2 mM group; ^Ψ^*P* < 0.05, vs. 5 mM group. Results are expressed as mean ± SEM.

We evaluated the potential effect of aspirin treatment on molecular consequences in SNK-6 cells. We first measured the effect of aspirin treatment on oxidative stress. The results showed that 5 and 10 mM of aspirin increased ROS formation to 168 and 187%, respectively (see Figure 3A), and increased 3-nitrotyrosine formation to 149 and 167%, respectively (see Figure 3B), while 1 and 2 mM of aspirin had no significant effects. We also measured the effect of aspirin on DNA damage. The results showed that 5 and 10 mM of aspirin increased 8-OHdG formation to 195 and 219%, respectively, while 1 and 2 mM of aspirin showed no effect (see Figure 3C). On the other hand, 2, 5, and 10 mM of aspirin increased γH2AX formation to 168, 206, and 253%, respectively (see Figures 3D,E). We then measured the effect of aspirin on mitochondrial function. The results showed that 2, 5 and 10mM of aspirin decreased ATP intracellular levels by 18, 40, and 57%, respectively (see Figure 3F), while 5 and 10mM of aspirin decreased mitochondria membrane potential (ΔΨm) by 35 and 43%, respectively (see Figure 3G). We finally measured the effect of aspirin on apoptosis in SNK-6 cells. We found that 5 and 10 mM of aspirin increased caspase-3 activity to 202 and 253%, respectively (see Figure 3H), and increased apoptosis rate to 320 and 390%, respectively (see Figures 3I,J). Our results indicate that higher doses of aspirin (5 and 10 mM) could achieve significant molecular consequences in SNK-6 cells, while lower doses (1 and 2 mM) of aspirin have little effect.

### Chidamide (CDM) Treatment Alone Slightly Suppresses VEGF Expression, Increases ROS Formation and Apoptosis, and Slightly Increases EBV Replication in SNK-6 Cells

In this study, we show that aspirin induces VEGF suppression and oxidative stress in NKTCL cells. It has been previously reported that chidamide induces oxidative stress (18, 20, 35) and VEGF signaling pathway in cancer cells (21). If chidamide could achieve the similar effect in NKTCL cells, this would point to the possibility that a combination of aspirin and chidamide may exert a synergistic effect in inducing VEGF suppression and oxidative stress in NKTCL cells. In Figure S3, we evaluated the potential effect of histone deacetylase inhibitor chidamide on SNK-6 cells. The results showed that 3 and 4 μM of chidamide decreased VEGF expression by 29 and 32%, respectively. In addition, they increased ROS formation to 134 and 147%, respectively (see Figure S3B), and also increased apoptosis rate to 219 and 245%, respectively (see Figure S3C) compared to the 0 μM treatment. On the other hand, 1 and 2 μM of chidamide showed no effect on NKTCL cells. We then evaluated the effect of chidamide on EBV virus in SNK-6 cells. The results showed that 3 and 4 μM of chidamide increased BZLF1 mRNA expression to 168 and 159%, respectively. It also increased BMRF1 mRNA expression to 156 and 169%, respectively (see Figure S3D) compared to 0 μM chidamide treatment, while 1 and 2 μM of chidamide had no effect on NKTCL cells. We also measured the effect of chidamide on EBV replication. The results showed that 2 μM of chidamide slightly increased EBV DNA copies to 128%, while other doses showed no effect. The results indicate that chidamide alone has small effects on SNK-6 cells.

### Aspirin Treatment Increases ROS Generation While Suppressing VEGF Expression and EBV Replication in SNK-6 Cells. Chidamide Significantly Potentiates This Effect

**Figure 4:**
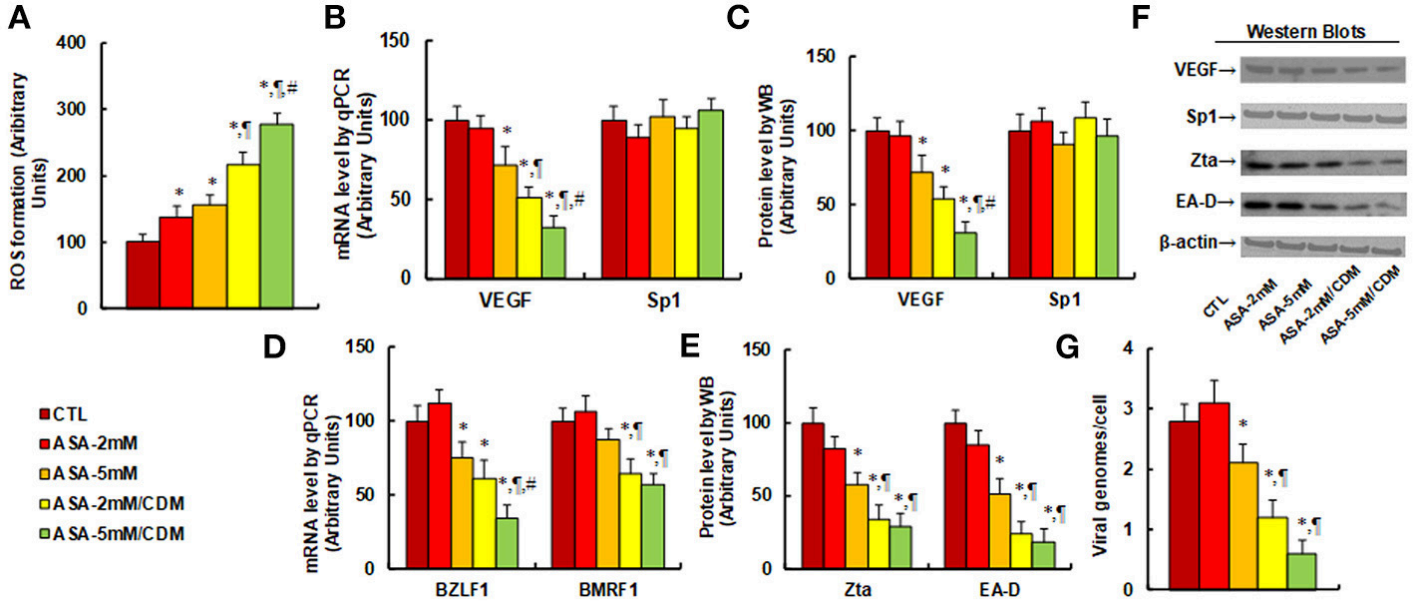
Aspirin treatment increases ROS generation and suppresses VEGF expression and EBV replication in SNK-6 cells. Chidamide significantly potentiates this effect. The SNK-6 cells were treated with control (CTL) alone, 2 mM aspirin (ASA) alone (ASA-2 mM), 5 mM ASA alone (ASA-5 mM), combination of 2 mM ASA and 3 μM CDM (ASA-2 mM/CDM), or combination of 5 mM ASA, and 3μM CDM (ASA-5mM/CDM) for 24 h, and the cells were harvested for further analysis. **(A)** ROS formation, *n* = 5. **(B)** mRNA levels by qPCR for VEGF and Sp1, *n* = 4. **(C)** Protein quantitation for VEGF and Sp1, *n* = 5. **(D)** mRNA level by qPCR for BZLF1 and BMRF1, *n* = 4. **(E)** Protein quantitation for Zta and EA-D, *n* = 5. **(F)** Representative pictures of western blots for **(C,E)**. **(G)** EBV viral genomes/cell by qPCR, *n* = 4. ^*^P < 0.05, vs. CTL group; ^¶^*P* < 0.05, vs. ASA-5 mM group; ^#^*P* < 0.05, vs. ASA-2 mM/CDM group. Results are expressed as mean ± SEM.

We first evaluated the effect of aspirin and chidamide on ROS generation. The results showed that aspirin treatment of 2 mM (ASA-2 mM) or 5 mM (ASA-5 mM) alone increased ROS generation to 137 and 156%, respectively, compared to the control (CTL) group. In addition, a combination of chidamide (CDM) with either 2 mM (ASA-2 mM/CDM) or 5 mM of aspirin (ASA-5 mM/CDM) further increased ROS generation to 216 and 278%, respectively (see Figure 4A). We then evaluated the effect of aspirin and chidamide on VEGF expression. The results showed that 2 mM of aspirin treatment (ASA-2 mM) alone had no effect, while 5 mM aspirin (ASA-5 mm) decreased VEGF mRNA levels by 28%. On the other hand, a combination of chidamide (CDM) with either 2 mM (ASA-2 mM/CDM) or 5 mM of aspirin (ASA-5 mM/CDM) significantly decreased VEGF mRNA levels by 49 and 68%, respectively, and had no effect on Sp1 expression (see Figure 4B). We also measured protein expression (see Figures 4C,F), and found that treatments of ASA-5 mM, ASA-2 mM/CDM and ASA-5mM/CDM decreased VEGF protein levels by 29, 46, and 69%, respectively, but had no effect on Sp1 expression. We then measured mRNA expression of BZLF1 and BMRF1, which is responsible for EBV activation. The results showed that treatments of ASA-5 mM, ASA-2 mM/CDM and ASA-5 mM/CDM decreased BDLF1 mRNA levels by 29, 46, and 69%, respectively, and treatments of ASA-2mM/CDM and ASA-5 mM/CDM decreased BMRF1 mRNA levels by 36 and 43%, respectively, while ASA-5 mM treatment had no effect (see Figure 4D). We also measured the related protein levels of those genes, including Zta and EA-D. The results showed that treatments of ASA-5 mM, ASA-2 mM/CDM and ASA-5 mM/CDM decreased Zta protein levels by 42, 66, and 71%, respectively, and decreased EA-D protein levels by 49, 76, and 82%, respectively (see Figures 4E,F). Finally, we measured the effect of aspirin and chidamide on EBV replication (see Figure 4G). We found that treatments of ASA-5 mM, ASA-2 mM/CDM and ASA-5 mM/CDM decreased EBV genome copies by 25, 57, and 79%, respectively. Our results indicate that ASA treatment alone slightly suppresses VEGF expression and EBV replication, while a combination with chidamide significantly potentiates this effect.

### Aspirin Treatment Suppresses Cell Proliferation, and Chidamide Significantly Potentiates This Effect in SNK-6 Cells

**Figure 5:**
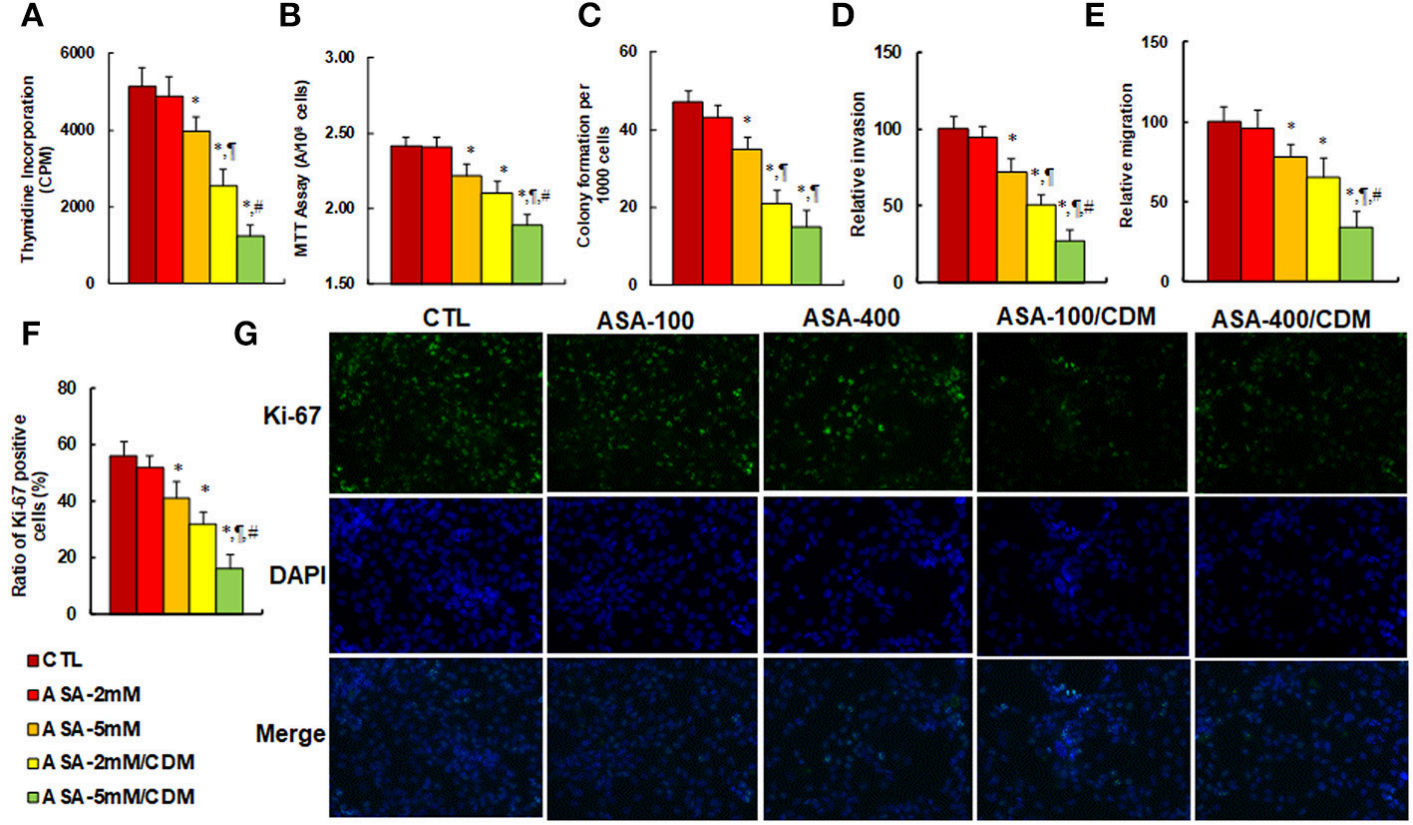
Aspirin treatment suppresses cell proliferation, and chidamide significantly potentiates this effect in SNK-6 cells. The SNK-6 cells were treated with control (CTL) alone, 2 mM aspirin (ASA) alone (ASA-2 mM), 5 mM ASA alone (ASA-5 mM), combination of 2 mM ASA and 3 μM CDM (ASA-2 mM/CDM), and combination of 5 mM ASA and 3 μM CDM (ASA-5 mM/CDM) for 24 h, and the cells were harvested for further analysis. **(A)** Cell proliferation analysis by thymidine incorporation, *n* = 5. **(B)** Cell metabolic activity by MTT assay, *n* = 5. **(C)** Colony formation assay in soft agar, *n* = 5. **(D)** Cell migration assay, *n* = 4. **(E)** Cell invasion assay, *n* = 4. **(F)** Quantitation of Ki-67 positive cells, *n* = 3. **(G)** Representative picture for **(F)**. ^*^P < 0.05, vs. CTL group; ^¶^*P* < 0.05, vs. ASA-5 mM group; ^#^*P* < 0.05, vs. ASA-2 mM/CDM group. Results are expressed as mean ± SEM. Results are expressed as mean ± SEM.

We evaluated the effect of aspirin and chidamide on *in vitro* tumor cell growth in SNK-6 cells. We first measured the effect of aspirin on thymidine incorporation assay. The results showed that treatments of ASA-5 mM, ASA-2 mM/CDM and ASA-5 mM/CDM decreased thymidine incorporation by 22, 50, and 76%, respectively (see Figure 5A) compared to the control (CTL) group, and decreased MTT assay by 8.0, 13, and 22%, respectively (see Figure 5B), in addition to decreasing colony formation by 26%, 55 and 68, respectively (see Figure 5C), while ASA-2 mM group showed no effect. We then measured the invasion and migration properties of the treated cells. We found that treatments of ASA-5 mM, ASA-2 mM/CDM and ASA-5 mM/CDM decreased cell invasion by 28, 49, and 73%, respectively (see Figure 5D) compared to the control (CTL) group, and that they decreased cell migration by 22, 35, and 66%, respectively (see Figure 5E). Finally, we measured the effect of aspirin on Ki-67 positive ratio using immunostaining. The results showed that treatments of ASA-5 mM, ASA-2 mM/CDM and ASA-5 mM/CDM decreased the Ki-67 positive ratio by 27, 43, and 71%, respectively (see Figures 5F,G), while the ASA-2 mM group showed no effect. Our results indicate that aspirin alone slightly decreases cell proliferation, and chidamide significantly potentiates this effect.

### Aspirin Suppresses Tumor Growth and EBV Replication in *in vivo* Xenograft Tumor Development, and Chidamide Significantly Potentiates This Effect

**Figure 6:**
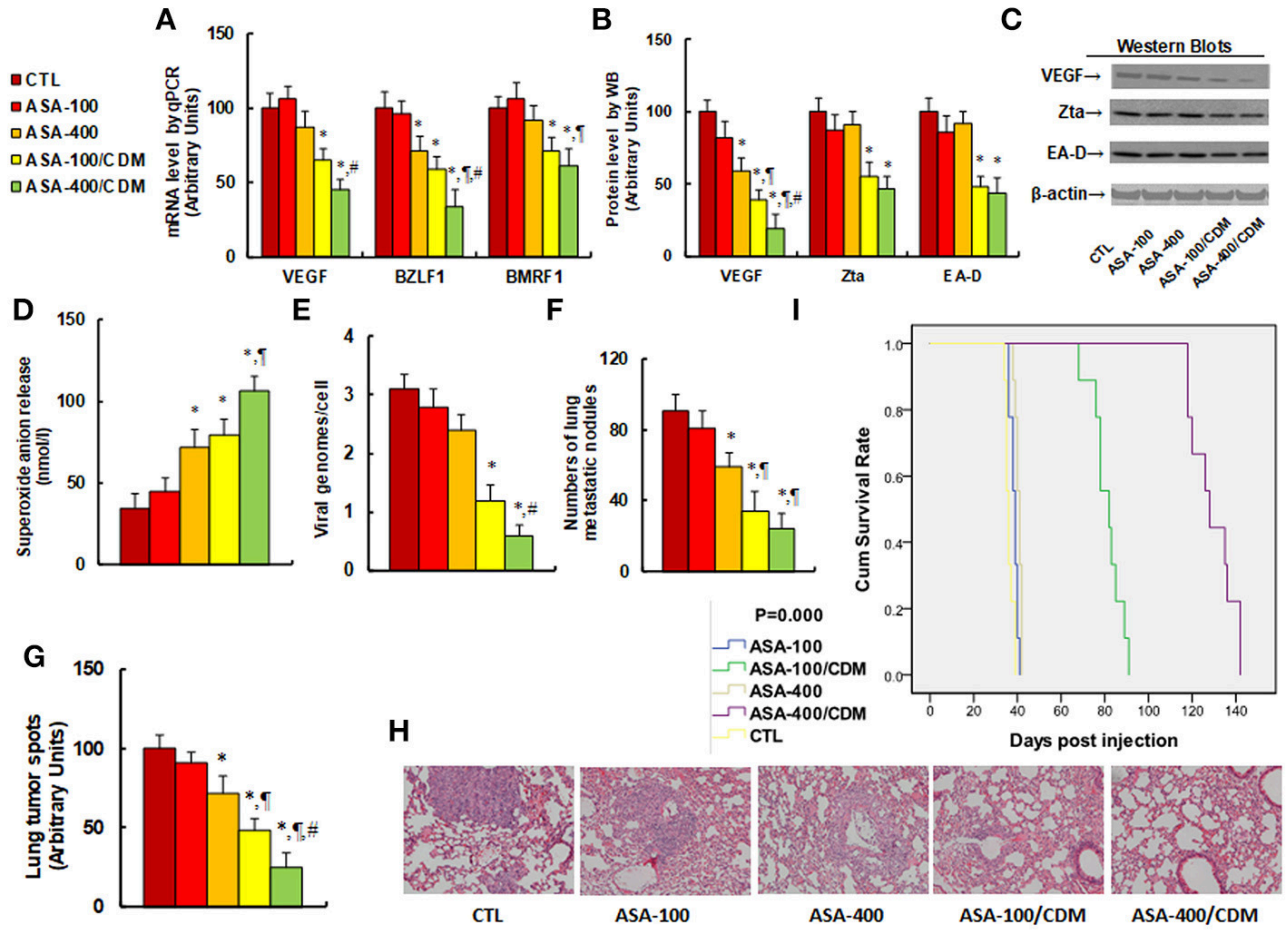
Aspirin suppresses tumor growth and EBV replication in *in vivo* xenograft tumor development, and chidamide significantly potentiates this effect. The nude mice were injected with SNK-6 cells for *in vivo* xenograft tumor development study. The 2 × 10^6^ viable treated tumor cells were washed, harvested in PBS, and then injected into the lateral tail vein in a volume of 0.1 ml. The mice were then treated with chemical control (CTL) alone, 100 mg/kg aspirin (ASA-100), 400 mg/kg aspirin (ASA-400), 100 mg/kg aspirin plus 25 mg/kg CDM (ASA-100/CDM), or 400 mg/kg aspirin plus 25 mg/kg CDM (ASA-400/CDM). Mice were monitored for changes in body weight and sacrificed when values fell below 20% of their initial weight for further analysis. **(A)** mRNA level by qPCR, *n* = 4. **(B)** Protein quantitation by Western Blots, *n* = 5. **(C)** Representative pictures for **(B)**. **(D)** Superoxide anion release from tumor tissues, *n* = 5. **(E)** EBV viral genomes/cell by qPCR, *n* = 5. **(F)** Tumor colony formation in lung, *n* = 5. **(G)** Mice were killed upon 20% weight loss, and organs were harvested for terminal analysis. Formalin-fixed, paraffin-embedded tumor tissue from the lungs were sectioned to 4 mm thickness, and the histopathological analyses were performed with H&E staining. Images were taken using a Carl Zeiss MIRAX MIDI slide scanner, and the lung tumor spots were analyzed using a 3DHISTECH Pannoramic Viewer, *n* = 5. **(H)** Representative picture by **H**&**E** staining for **(G)**. **(I)** Kaplan-Meier analysis comparing survival of mice between each treatment group, *P-*value represents log-rank Mantel-Cox test result, *n* = 10. ^*^P < 0.05, vs. CTL group; ^¶^*P* < 0.05, vs. ASA-400 group; ^#^*P* < 0.05, vs. ASA-100/CDM group. Results are expressed as mean ± SEM.

We evaluated the effect of aspirin on tumor growth and EBV replication in *in vivo* xenograft tumor tissues. The 2 × 10^6^ viable treated tumor cells were washed, harvested in PBS, and then injected into the lateral tail vein in a volume of 0.1 ml. Mice were monitored for changes in body weight and sacrificed when values fell below 20% of their initial weight. We first measured the effect of aspirin on gene expression. The results showed that treatments of ASA-100/CDM and ASA-400/CDM decreased VEGF mRNA levels by 35 and 55%, respectively, and they decreased BMRF1 mRNA levels by 29 and 39%, respectively, compared to the control (CTL) group. Furthermore, treatments of ASA-400, ASA-100/CDM and ASA-400/CDM decreased BZLF1 mRNA levels by 29, 41, and 66%, respectively (see Figure 6A). We then measured the protein levels of these genes. The results showed that treatments of ASA-400, ASA-100/CDM and ASA-400/CDM decreased VEGF by 41, 61, and 81%, respectively, and treatments of ASA-100/CDM and ASA-400/CDM decreased Zta by 45 and 53%. They also decreased EA-D by 52 and 56%, respectively, while treatment of the ASA-400 group had no effect (see Figures 6B,C). We measured the effect of aspirin on oxidative stress in the tumor tissues. The results showed that treatments of ASA-400, ASA-100/CDM, and ASA-400/CDM increased superoxide anion (O${}_{2}^{-.}$) *in vivo* release to 212, 232, and 312%, respectively, compared to the control group (see Figure 6D). We also measured the EBV DNA copies in the tumor tissues. We found that treatments of ASA-100/CDM and ASA-400/CDM decreased EBV DNA copies by 61 and 81%, respectively, compared to the control group (see Figure 6E). We then measured lung tumor nodule formation from the tumor tissues and found that treatments of ASA-400, ASA-100/CDM, and ASA-400/CDM decreased lung tumor nodule formation by 35, 63, and 74%, respectively, compared to the control group (see Figure 6F). Additionally, we evaluated the effect of aspirin on the formation of lung tumor spots by H&E staining (see Figures 6G,H). The results showed that treatments of ASA-400, ASA-100/CDM, and ASA-400/CDM decreased lung tumor spots by 28, 52, and 75%, respectively. Finally, we measured the effect of aspirin on mouse survival rate using Kaplan-Meier analysis (see Figure 6I). We found that treatment of aspirin alone, including the ASA-100 and ASA-400 group, had little effect on mouse survival, while treatments with the addition of chidamide, including the ASA-100/CDM and ASA-400/CDM groups, resulted in significantly increased mouse survival - to 225 and 358% compared to the control (CTL) group, respectively. Our results indicate that aspirin alone has a small effect on tumor growth and EBV replication, while a combination of aspirin and chidamide significantly potentiates aspirin-mediated *in vivo* tumor growth and EBV suppression.

## Discussion

In this study, we demonstrated that aspirin inhibits VEGF expression through histone methylation with subsequently decreased association of Sp1 on the VEGF promoter. In addition, aspirin modulates mitochondrial function and increases ROS generation with subsequent suppression of EBV replication. This is a potential mechanism of aspirin in suppression of EBV-associated NKTCL tumor growth. Furthermore, we found that the suppression effect of aspirin on NKTCL tumor growth is significantly potentiated with the addition of chidamide. This provides a new strategy for the clinical treatment of NKTCL.

### Aspirin-Mediated VEGF Suppression and Tumor Suppression

In this study, we showed that aspirin inhibits NKTCL tumor growth through VEGF suppression, which is consistent with previous reports on the effects of aspirin on tumor suppression (5, 7, 8). It has been reported that histone methylation is involved with VEGF expression (36), and that Sp1 is involved in VEGF expression and tumor growth (37). Our results showed that aspirin increases histone methylation on the promoter of VEGF (12), and subsequently decreases the binding ability of Sp1 to the VEGF promoter in NKTCL cells, while Sp1 expression does not change. On the other hand, other reports have shown that aspirin inhibits tumor growth through direct downregulation of Sp1 in colon cancer cells (33); this discrepancy may be due to different gene regulation systems of Sp1 in different cells. Furthermore, we found that aspirin-induced VEGF expression is involved in H3K27me3 histone modification. It has been reported that polycomb repressive complex 2 (PRC2) is responsible for methylation of histone H3 on lysine 27 (H3K27me3) (38), and that PRC2 comprises the EZH1/2 catalytic subunit, SUZ12, EED, and RBBP7/4 (39). Either knockdown of the EZH2 subunit by siRNA or the EED specific inhibitor EED226 (34) should be able to block PRC2-induced H3K27me3 methylation. Our results showed that treatments of both EZH2 siRNA and EED specific inhibitor EED226 completely restored aspirin-induced H3K27me3 modification and VEGF suppression. This provides evidence that aspirin-induced VEGF suppression is involved in H3K27me3 modification on the VEGF promoter.

### Aspirin-Mediated Mitochondrial Function and EBV Inhibition

It has been reported that aspirin modulates ROS generation (40) and mitochondrial function (15, 41). In this study, we showed that aspirin increases oxidative stress and subsequent DNA damage. Furthermore, it decreases ATP generation and mitochondrial membrane potential with subsequently increased apoptosis, which is consistent with previous reports. The potential mechanism of aspirin in the modulation of mitochondrial function is complicated; it can be explained by speculations that aspirin may modulate mitochondrial biogenesis (42), increase mitochondrial fatty acid oxidation (43), or modulate mitochondrial voltage-dependent anion channels (VDAC) (44). In addition, our data showed that aspirin slightly suppresses EBV replication and decreases viral gene expression of BZLF1 and BMRF1 and the subsequent coding proteins Zta and EA-D (45, 46). BZLF1 and BMRF1 are potential markers of EBV activation during the latent stage, and the suppression of Zta and EA-D may indicate a potential effect of aspirin on EBV DNA removal. This can be explained because aspirin-mediated mitochondrial dysfunction and ROS generation triggers EBV DNA damage, subsequently suppressing EBV replication, which is consistent with our previous findings (20).

### Role of Chidamide in Aspirin-Mediated Tumor Suppression

As a novel histone deacetylase inhibitor, chidamide (CDM) has been recently used for several clinical trials as a potential anti-tumor drug in China. It shows some effects on cancer inhibition in T-cell lymphoma (19, 47–49), multiple myeloma (50), and pancreatic cancer (51), although there are no reports regarding the effects of chidamide alone on NKTCL inhibition. In this study, we showed that chidamide (CDM) alone has a small effect on NKTCL tumor growth and EBV replication, while when it is used together with aspirin, it significantly potentiated aspirin-mediated tumor suppression in NKTCL cells. NKTCL is an EBV-associated tumor with a tendency to be relapsed or refractory and is resistant to many chemotherapies, and a combination of aspirin and chidamide (ASP/CDM)-mediated ROS over-generation could directly bring damage to EBV DNA and inhibit EBV replication (20). Furthermore, ASP/CDM-mediated VEGF pathway suppression could significantly suppress tumor growth (21). Our data provides a new strategy for NKTCL treatment through suppression of the VEGF signaling pathway and ROS generation.

## Conclusions

**Figure 7:**
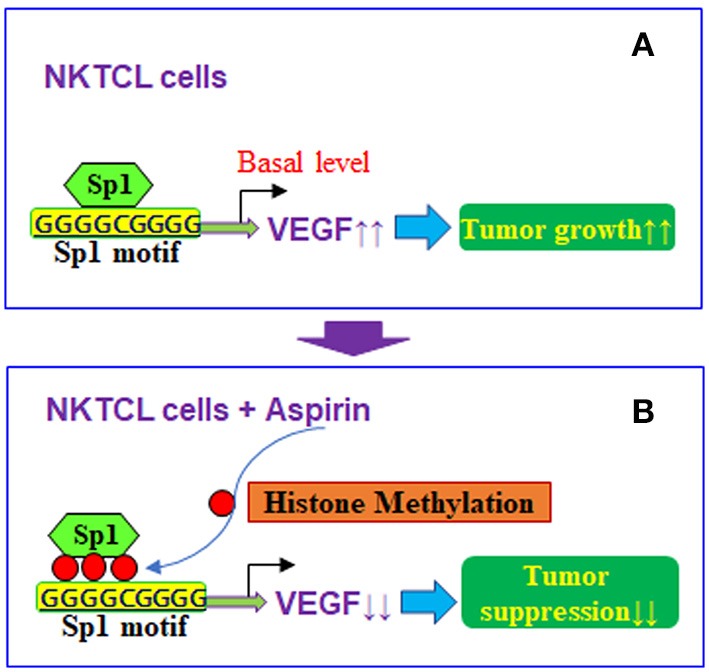
Proposed mechanisms for aspirin-mediated VEGF suppression in NKTCL cells. **(A)** VEGF expression in NKTCL cells with tumor growth. **(B)** VEGF suppression by aspirin-mediated histone methylation and subsequent Sp1 dissociation on the VEGF promoter in NKTCL cells with tumor suppression.

Taken together, aspirin-mediated suppression of VEGF expression and tumor growth in NKTCL cells can be conceptualized in Figure 7. We showed that aspirin suppresses VEGF expression through epigenetic changes on the VEGF promoter with increased histone methylation. Furthermore, it modulates mitochondrial function with increased ROS formation and apoptosis in NKTCL tumor cells. Aspirin alone slightly inhibits NKTCL tumor growth, while this effect is significantly potentiated with the addition of the histone deacetylase inhibitor chidamide. We conclude that aspirin inhibits NKTCL through VEGF suppression and modulation of mitochondrial formation. The addition of chidamide significantly potentiates aspirin-mediated NKTCL inhibition. This provides a new strategy for anti-tumor drug development to inhibit EBV-associated NKTCL tumor growth.

## Ethics Statement

The animal protocol conformed to US NIH guidelines (Guide for the Care and Use of Laboratory Animals, No. 85-23, revised 1996), and was reviewed and approved by the Institutional Animal Care and Use Committee from Peking University Shenzhen Hospital and Wuhan University.

## Author Contributions

PY wrote the paper. PY, LL, and WX designed, analyzed the data and interpreted the experiments. ML, AY, XH, and WX performed vector constructions and gene expression analysis. QG and ZW performed statistical analysis and part of the mice experiments. QC and ZY performed gene analysis and part of the mapping analysis. HZ, JL, and YJ performed the remaining experiments. All authors read and approved the final manuscript.

### Conflict of Interest Statement

The authors declare that the research was conducted in the absence of any commercial or financial relationships that could be construed as a potential conflict of interest.

## Abbreviations

ASA: acetylsalicylic acid (Aspirin)
CDM: chidamide
ChIP: chromatin Immunoprecipitation
EBV: Epstein-Barr virus
HDAC: Histone deacetylase
HDACi: histone deacetylase inhibitors
NKTCL: Extranodal nasal-type natural killer/T-cell lymphoma
O2: superoxide anions
ROS: reactive oxygen species
Sp1: specificity protein 1
VEGF: vascular endothelial growth factor.
