# Supplementary material for: Aspirin Inhibits Natural Killer/T-Cell Lymphoma by Modulation of VEGF Expression and Mitochondrial Function
Source: Front Oncol. 2019 Jan 14;8:679. doi: 10.3389/fonc.2018.00679 (PMC6339948; doi:10.3389/fonc.2018.00679)
Supplement: Supplementary file 1 [file Table_1.docx]

**Aspirin Inhibits Natural Killer/T-Cell Lymphoma by Modulation of VEGF Expression and Mitochondrial Function**

Hongyu Zhang^1,^*, Jianping Lu^2,^*, Yun Jiao^3,^*, Qi Chen^1^, Min Li^4^, Zichen Wang^2^, Zhendong Yu^1^, Xiaodong Huang^4^, Athena Yao^4^, Qiong Gao^5^, Weiguo Xie^4,#^, Ling Li^3,#^, Paul Yao^1,2,3,4,#^

**Supplementary Materials**

**Table S1. Sequences of primers for the real time quantitative PCR (qPCR)**

| Gene | Species | Analysis | Forward primer (5'→3') | Reverse primer (5'→3') |
| --- | --- | --- | --- | --- |
| β-actin | Human | mRNA | gatgcagaaggagatcactgc | atactcctgcttgctgatcca |
| Sp1 | Human | mRNA | taccttgactcccagctctca | gctattggcattggtgaaaaa |
| VEGF | Human | mRNA | gccagcacataggagagatga | catttacacgtctgcggatct |
| EED | Human | mRNA | aagcagaagctgagcagtgac | aacttttccttccaggtgcat |
| EZH2 | Human | mRNA | gatgatggagacgatcctgaa | cttctgctgtgcccttatctg |
| BZLF1 | EBV | mRNA | gggggataatggagtcaacat | tagcgtcccaaacataaatgc |
| BMRF1 | EBV | mRNA | tcctgtccaagtgctatgacc | gggagacctcgaagctgatta |
| β-actin | Human | Genome | ctggacttcgagcaagagatg | aggaaggaaggctggaagagt |
| BMRF1 | EBV | Genome | ccgtcctgtccaagtgctat | gggagacctcgaagctgatta |
| VEGF | Human | ChIP | gggagccagagaccagtg | cccaaaacttttcccaaactc |

FIGURE S1

**Figure S1. Aspirin treatment suppresses VEGF expression in NKTCL cells.** Different kinds of NKTCL cells were treated by ASA with different concentrations of 0, 1, 2, 5 and 10mM for 24 hours, and then the cells were used for mRNA analysis by qPCR. (a) HANK-1 cells, n=4. (b) NK-92 cells, n=4. (c) SNT-8 cells, n=4. *, *P*<0.05, vs CTL group. Data are expressed as mean ± SEM.

FIGURE S2

**Figure S2. Aspirin-mediated epigenetic changes on the VEGF promoter in SNK-6 cells.** SNK-6 cells were treated by aspirin (ASA) with different concentrations of 0, 1, 2, 5 and 10mM for 24 hours, and then the cells were used for ChIP analysis. (a) Aspirin-mediated histone acetylation on the VEGF promoter using H3K9,14,18,23,27ac and H4K5,8,12,16ac antibodies. (b) Aspirin-mediated histone H4 methylation on the VEGF promoter. n=5.

FIGURE S3

**Figure S3. Chidamide (CDM) treatment alone slightly suppresses VEGF expression, increases ROS formation and apoptosis, and slightly increases EBV replication in SNK-6 cells.** The SNK-6 cells were treated with different concentrations of chidamide (CDM) for 24 hours, and the cells were harvested for analysis. (a) VEGF mRNA level by qPCR, n=4. (b) ROS formation, n=5. (c) Apoptosis rate by TUNEL assay, n=5. (d) mRNA levels for BZLF1 and BMRF1, n=4. (e) EBV viral genomes/cell by qPCR, n=4. *, P<0.05, vs 0 μM Chidamide. Results are expressed as mean ± SEM.

FIGURE S4

**Figure S4. Effect of VEGF/chidamide(CDM) treatment on HANK-1 and MNCs cells.** Either HANK-1 or MNCs cells were treated with control (CTL) alone, 2mM aspirin (ASA) alone (ASA-2mM), 5mM ASA alone (ASA-5mM), combination of 2mM ASA and 3μM CDM (ASA-2mM/CDM), and combination of 5mM ASA and 3μM CDM (ASA-5mM/CDM) for 24 hours, and the cells were harvested for further analysis. (a) ROS formation, n=5. (b) mRNA level by qPCR for VEGF, n=4. (c) Apoptosis rate by TUNEL assay, n=5. (d) Quantitation of Ki-67 positive cells, n=3. (e) mRNA level by qPCR for BZLF1 and BMRF1, n=4. *, *P*<0.05, vs CTL group; ¶, *P*<0.05, vs ASA-5mM group; #, *P*<0.05, vs ASA-2mM/CDM group. Results are expressed as mean ± SEM.
